# Thyroid Remnant Estimation by Diagnostic Dose ^131^ **I** Scintigraphy or ^99**m**^ **T** **c** **O**_4_^−^ Scintigraphy after Thyroidectomy: A Comparison with Therapeutic Dose ^131^ **I** Imaging

**DOI:** 10.1155/2016/4763824

**Published:** 2016-01-21

**Authors:** Guanghui Liu, Na Li, Xuena Li, Song Chen, Bulin Du, Yaming Li

**Affiliations:** Department of Nuclear Medicine, The First Hospital of China Medical University, Shenyang 110001, China

## Abstract

In this clinical study, we have compared routine diagnostic dose ^131^I scan and ^99m^TcO_4_^−^ thyroid scintigraphy with therapeutic dose ^131^I imaging for accurate thyroid remnant estimation after total thyroidectomy. We conducted a retrospective review of the patients undergoing total thyroidectomy for differentiated thyroid carcinoma (DTC) and subsequently receiving radioactive iodine (RAI) treatment to ablate remnant thyroid tissue. All patients had therapeutic dose RAI whole body scan, which was compared with that of diagnostic dose RAI, ^99m^TcO_4_^−^ thyroid scan, and ultrasound examination. We concluded that therapeutic dose RAI scan reveals some extent thyroid remnant in all DTC patients following total thyroidectomy. Diagnostic RAI scan is much superior to ultrasound and ^99m^TcO_4_^−^ thyroid scan for the postoperative estimation of thyroid remnant. Ultrasound and ^99m^TcO_4_^−^ thyroid scan provide little information for thyroid remnant estimation and, therefore, would not replace diagnostic RAI scan.

## 1. Introduction

Thyroid carcinoma is one of the most common malignant tumors in the endocrine system; the estimated incidence of thyroid cancer currently was around 0.01% to 0.03% per year [1]. Differentiated thyroid carcinoma (DTC) accounts for 90% of all thyroid cancers [2].

DTC treatment guidelines recommended a near total or total thyroidectomy for patients with tumor size greater than 1 cm in diameter or those who have multiple lesions or distal metastases. Radioiodine (RAI) remnant ablation has been increasingly used to eliminate the postsurgical thyroid remnant [3].

The remnant thyroid volume affects the ablation dose of RAI [4, 5], so it was important to identify remnant volume for further treatment after surgery. The extent of remnant thyroid plays a significant role for DTC recurrence and the evaluation of remnant thyroid with imaging technique may provide additional information for prediction of the recurrence and also for estimation of the amount of radioiodine to be used for ablation.

^99m^TcO_4_^−^ scintigraphy, routine diagnostic radioiodine whole body scintigraphy, and ultrasound have been widely used to estimate thyroid remnant following thyroidectomy. However which is the ideal imaging modality for accurate thyroid remnant estimation after total thyroidectomy was not well concluded.

Thyroglobulin (Tg) is glycoprotein secreted by thyroid follicular epithelial cells with plasma half-life of 3.7 h to 4.3 d [6]. Thyroglobulin antibody (TgAb) is the antibody of Tg. Both of them are regulated by thyroid-stimulating hormone (TSH). The serum levels of Tg, TgAb, and TSH have been used as indicators for thyroid remnant [7–9]. In this study, we also observed these parameters and related to thyroid remnant defined by RAI scan.

In this retrospective analysis, we have compared routine diagnostic dose radioiodine whole body scintigraphy, ^99m^TcO_4_^−^ thyroid scan, and ultrasound with that post-Iodine-131 ablation therapeutic dose imaging.

## 2. Material and Methods

### 2.1. Patients

This study was preapproved by the hospital institutional review boards of the First Hospital of China Medical University (Shenyang, China). This retrospective analysis used the following inclusion criteria: total thyroidectomy, no lymph node metastasis or distant metastasis, and the first time of RAI ablation. A total of 100 DTC patients from October 2011 to August 2014 were included; all of them had total thyroidectomy with pathologically confirmed as thyroid papillary carcinoma and were subsequently referred to our department for the first time of RAI remnant ablation. The patients' age ranged from 13 to 71 years (mean 43 years), 76 of them were female and 24 were male. The duration from thyroidectomy to RAI ablation was 1–12 months (mean 4 months). Before RAI ablation, all patients fasted from iodine-enriched foods and medications (sea food, milk products, and iodine containing ointments/balms) for 2–4 weeks. Patients had either ^99m^TcO_4_^−^ thyroid scan or diagnostic RAI whole body scan (WBS) or ultrasound (US) examination prior to ^131^I ablation. All patients had RAI whole body scans 3 days after therapeutic dose of ^131^I administration.

### 2.2. Imaging Protocols

For ^99m^TcO_4_^−^ scan, the patients were intravenously injected with ^99m^TcO_4_^−^ 185 MBq (5 mCi) and after 30 mins, anterior planar thyroid scan was acquired for 5 min using a Symbia T2 SPECT/CT detector (Siemens, Germany) equipped with low-energy and high-resolution collimators (matrix 256 × 256, Zoom 2), the peak energy is 140 keV, and window width was set as 20%.

For diagnostic dose RAI whole body scan, the patient took 74 MBq (2 mCi) ^131^I (NaI) solution orally after overnight fasting and whole body scan was performed 24 hours later using a Symbia T2 SPECT/CT detector equipped with high-energy collimators (matrix 128 × 128, Zoom 1). The scan speed was set as 15 cm/min.

For therapeutic dose RAI whole body imaging, the patients took ^131^I 3.7 GBq (100 mCi) orally after overnight fasting for ablate thyroid remnant. Whole body scan was performed 3 days later, whole body scan was done following above-mentioned diagnostic ^131^I scan protocol and imaging was obtained from the same device.

Three board-certified nuclear medicine attending physicians read RAI whole body imaging and ^99m^TcO_4_^−^ thyroid scan. Negative result was defined as tracer uptake was not exceeding neck background. Each modality imaging was compared with therapeutic dose RAI whole body scan which served as a “gold standard” for defining the presence or absence of remnant thyroid. Severe remnant was defined as imaging agent uptake in the thyroid bed being strong, imaging appearing as “sunshine” shape; mild remnant group as imaging agent uptake in the thyroid bed being weaker, with no “sunshine” appearance; and no remnant defined as no tracer uptake beyond neck background in the thyroid bed [10].

### 2.3. Serological Examination

The serological examination of Tg, TgAb, and TSH was taken in our hospital 1–3 days before the patients took therapeutic dose ^131^I.

### 2.4. Statistical Analysis

Statistical analysis was performed by SPSS 17.0; a *p* value less than 0.05 was considered as statistically significant.

## 3. Results

**Figure 1:**
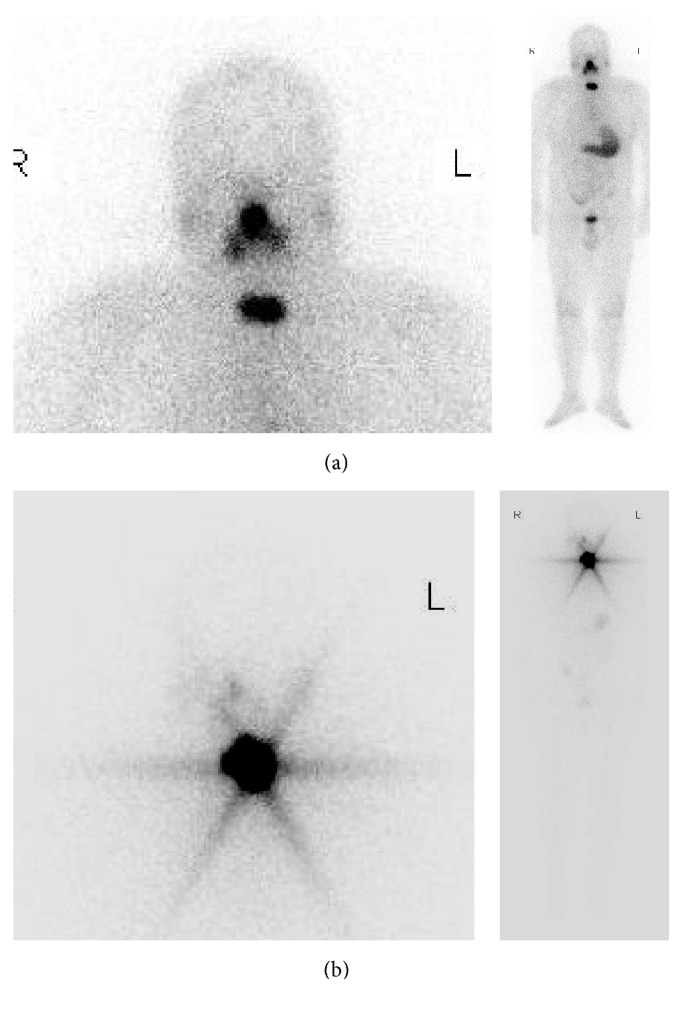
Extent of thyroid remnant detected after ^131^I ablation imaging (therapeutic RAI whole body scan). (a) Mild remnant of a 37-year-old male after total thyroidectomy. Whole body scan and regional image 3 days after 3.7 GBq (100 mCi) ^131^I oral administration. (b) Severe remnant of a 31-year-old female after total thyroidectomy. Whole body scan and regional image 3 days after 3.7 GBq (100 mCi) ^131^I oral administration.

Therapeutic dose RAI whole body scans revealed that all thyroid beds following total thyroidectomy contained residual thyroid tissue which accumulated at least some extent of ^131^I. Accordingly, of 100 patients, 55 patients had mild thyroid remnant and 45 had severe remnant. Representative therapeutic RAI images showed mild thyroid remnant (Figure 1(a)) and severe remnant (Figure 1(b)).

Of 100 patients, all had whole body scans three days after therapeutic iodine administration. Before RAI thyroid ablation, ultrasound and ^99m^TcO_4_^−^ thyroid scan were done in 45 patients and ultrasound and diagnostic dose ^131^I whole body scan were performed in 39 patients, while just ultrasound exam was conducted in 15 patients; the results were summarized in Table 1. Diagnostic RAI scan detected thyroid remnant in 67% (26/39) patients and ^99m^TcO_4_^−^ thyroid scan had a sensitivity of 13% (6/45), while as ultrasound the sensitivity was only 8% (8/99). The sensitivity for diagnostic RAI scan to detect thyroid remnant was significantly higher than ^99m^TcO_4_^−^ thyroid scan (*p* < 0.001) and ultrasound (*p* < 0.001) and there was no significant difference between ^99m^TcO_4_^−^ thyroid scan and ultrasound (*p* = 0.34).

**Figure 2:**
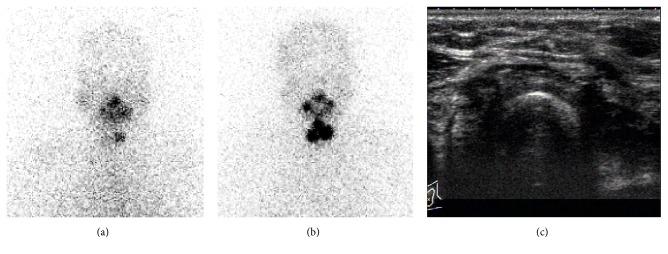
Comparing diagnostic ^131^I scan with postablation therapy ^131^I imaging. A 49-year-old female after total thyroidectomy. (a) Diagnostic RAI scan performed 24 hours after 74 MBq (2 mCi) ^131^I oral administration. (b) Therapeutic dose scan performed 3 days after 3.7 GBq (100 mCi) ^131^I oral administration 3 weeks after diagnostic ^131^I scan. Thyroid remnant is visualized by diagnostic and therapeutic dose of ^131^I scans, but smaller residual thyroid is found by diagnostic scan. (c) Ultrasound fails to detect thyroid remnant.

Figure 2 showed thyroid remnant was detected by both diagnostic dose and therapeutic dose RAI whole body scans in a 49-year-old female who underwent total thyroidectomy for treating DTC, though the extent thyroid remnant imaged by diagnostic RAI was apparently smaller than therapeutic RAI scan. Ultrasound failed to detect the presence of remnant thyroid tissue.

**Figure 3:**
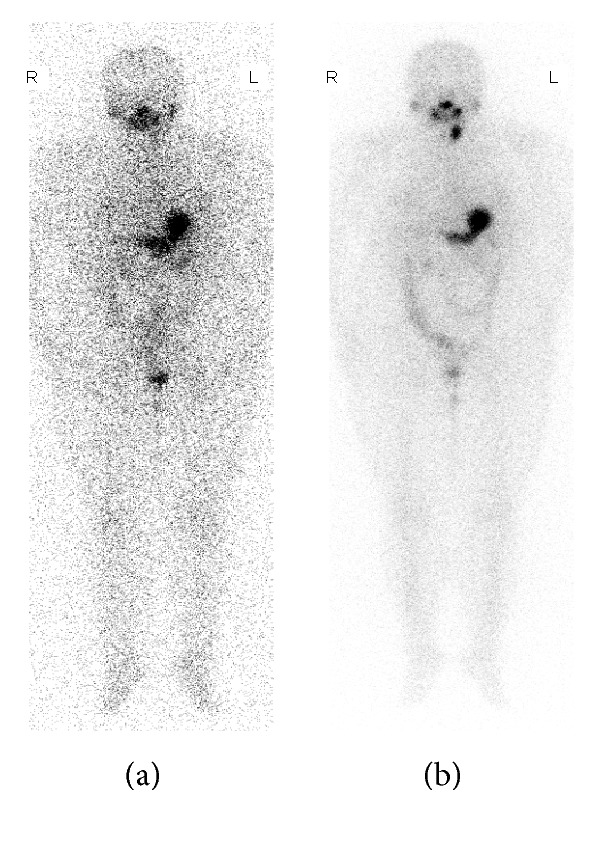
Difference between diagnostic and therapeutic dose ^131^I scan. A 37-year-old patient with total thyroidectomy. Diagnostic dose whole body scan (a) fails to visualize the presence of thyroid remnant identified by therapeutic dose of ^131^I scan (b).

**Figure 4:**
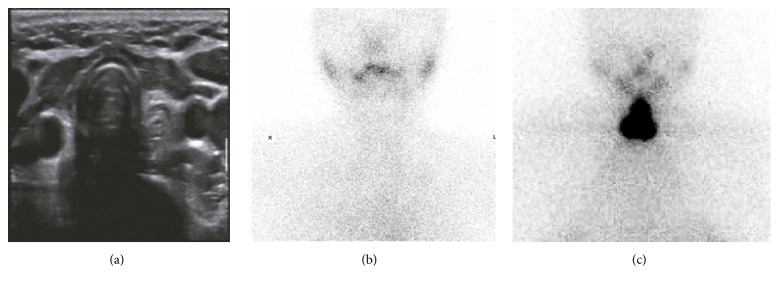
Comparing ^99m^TcO_4_^−^ thyroid scan and ultrasound therapeutic dose of ^131^I scan for thyroid remnant detection in the same patient. A 32-year-old female after total thyroidectomy. Ultrasound (a) and ^99m^TcO_4_^−^ thyroid scan (b) fail to visualize thyroid remnant detected by therapeutic dose of ^131^I scan (c).

On the other hand, 33% diagnostic dose RAI whole body scan failed to map thyroid remnant which was presented in diagnostic RAI imaging (Figure 3). ^99m^TcO_4_^−^ thyroid scan and ultrasound failed to image majority of thyroid remnant, an example of  ^99m^TcO_4_^−^ thyroid scan; ultrasound and diagnostic RAI scan were presented in Figure 4.

Serum levels of Tg, TgAb, and TSH between mild group and severe group were analyzed and summarized in Table 2. The levels of Tg and TgAb were significantly higher in severe remnant patients than mild remnant patients; therefore Tg and TgAb can be used as indicators of extent thyroid remnant. TSH values had no statistical difference between the two groups.

As the ability of diagnostic dose RAI whole body scan for detecting thyroid remnant is also dependent on TSH level, we analyzed the TSH level between the two groups of different diagnostic dose RAI whole body scan results and there was no significant difference (*t* = 1.475, *p* = 0.493).

## 4. Discussion

Although there has been considerable debate as to the role of total versus less than total thyroidectomy (usually lobectomy and isthmusectomy) for differentiated thyroid cancers [11, 12], most people suggest total thyroidectomy. Large databases showing statistically significant differences in outcome between total thyroidectomy and lobectomy for tumors >1 cm. Furthermore, these patients can then undergo RAI therapy and use Tg levels as a marker to monitor recurrent diseases [13]. The guidelines for DTC therapy recommend total thyroidectomy for cancer bigger than 1 cm in diameter. Thyroid remnant in DTC after thyroidectomy may contain micrometastases which may be the source of recurrence or metastasis [14].

A follow-up report of the impact of therapy in 576 patients showed a 45% risk of recurrence in total thyroidectomy only; the risk in total thyroidectomy and TSH suppression therapy and total thyroidectomy + TSH suppression therapy + radioiodine (RAI) remnant ablation were 11% and 2.7%, respectively [15]. Therefore estimation of thyroid remnant and the role of postoperative remnant ablation with RAI is important for curing DTC.

Our therapeutic dose RAI scan findings indicated that thyroid remnant existed in all patients after total thyroidectomy (Figure 1). This is not a surprise: surgeons leave some thyroid tissue near the upper parathyroid and the insertion of the recurrent laryngeal nerve to protect these structures; this is so called near total thyroidectomy [16].

Our data clearly demonstrated that, in addition to therapeutic dose RAI whole body scan, diagnostic RAI scan had far higher sensitivity for thyroid remnant evaluation (Table 1). Although ultrasound and ^99m^TcO_4_^−^ thyroid scan are routinely used in thyroid clinic, both strategies provided minimal information with respect to thyroid remnant (Table 1, Figure 4). Therefore, our results suggested not to use ultrasound and ^99m^TcO_4_^−^ thyroid scan for thyroid remnant estimation.

^99m^TcO_4_^−^ is a widely used tracer for thyroid imaging in clinical setting. ^99m^TcO_4_^−^ scan reported a sensitivity of 87%, specificity of 97%, and accuracy of 92.5% for detecting thyroid remnant and ectopic thyroid tissue when compared to ^131^I scan [17].

However, our data indicated that ^99m^TcO_4_^−^ thyroid scans provided little information to detect thyroid remnant, which was far worse than ^131^I (Figure 4, Table 1). Therefore, ^99m^TcO_4_^−^ thyroid scan should be carefully considered to locate thyroid remnant.

Compared to other modalities, the coincidence rate between diagnostic RAI scan and therapeutic RAI imaging was much higher in identifying thyroid remnant. However, we recognize that the difference between diagnostic dose and therapeutic dose scan was apparent (Table 1, Figures 2 and 3), this may be due to the difference in amount of administration dose of ^131^I (100 mCi vs. 2 mCi), as well as the time of scans (3 d vs. 1 d). Of note, negative diagnostic RAI imaging frequently indicated mild thyroid remnant.

The levels of Tg, in theory, could not be detected after being fully removed from thyroid tissue. We found that Tg still could be detected 12 months after so called total thyroidectomy; the levels of Tg as well as TgAb in severe group (defined by therapeutic RAI scan) were significantly higher than mild group. Therefore, the serum level of Tg also had a certain value in the evaluation of the degree of postoperative thyroid remnant.

About 10%~40% DTC patients after total thyroidectomy and ^131^I ablation had detectable serum level of TgAb [18, 19], which is probably due to the following reasons: DTC patients might still have memory lymphocyte after treatment, they maintain the ability to produce TgAb; the radiation damage of RAI remnant ablation causes the release of antigen; and metastases have the ability to produce TgAb which become the source of autoantigen. Elevated levels of serum TgAb may also be an indicator of thyroid remnant, but it seems less specific than Tg.

## 5. Conclusions

Therapeutic dose RAI scan reveals the extent thyroid remnant in all DTC patients following total thyroidectomy. Diagnostic RAI scan is much superior to ultrasound and ^99m^TcO_4_^−^ thyroid scan for the postoperative estimation of thyroid remnant. Ultrasound and ^99m^TcO_4_^−^ thyroid scan provide little information for thyroid remnant estimation and, therefore, would not replace diagnostic RAI scan.

## Figures and Tables

**Table 1:** Comparison of ultrasound ^99m^TcO_4_^−^ thyroid scan and diagnostic dose ^131^I scan for thyroid remnant detection.

Imaging strategy	Positive	Negative	Total
Ultrasound	8	91	99
^99m^TcO_4_^−^ thyroid scan	6	39	45
Diagnostic dose ^131^I scan	26	13	39

**Table 2:** Serum TG, TGAb, and TSH level and extent thyroid remnant.

Serum level	Mild remnant	Severe remnant	*p*
TG (ng/mL)	4.00 ± 7.24	22.23 ± 52.68	0.000
TGAb (IU/mL)	25.16 ± 65.16	85.27 ± 241.44	0.001
TSH (mIU/mL)	63.82 ± 27.58	47.93 ± 25.92	0.400

Thyroglobulin (Tg); thyroglobulin antibody (TgAb); thyroid-stimulating hormone (TSH).
